# Fibrillar Amyloid Plaque Formation Precedes Microglial Activation

**DOI:** 10.1371/journal.pone.0119768

**Published:** 2015-03-23

**Authors:** Christian K. E. Jung, Kevin Keppler, Sonja Steinbach, Lidia Blazquez-Llorca, Jochen Herms

**Affiliations:** 1 Department for Translational Brain Research, German Center for Neurodegenerative Diseases—site Munich (DZNE-M) and Center for Neuropathology and Prion Research (ZNP), Ludwig-Maximilians-University Munich, Munich, Germany; 2 Munich Cluster of Systems Neurology (SyNergy), Munich, Germany; Massachusetts General Hospital and Harvard Medical School, UNITED STATES

## Abstract

In Alzheimer’s disease (AD), hallmark β-amyloid deposits are characterized by the presence of activated microglia around them. Despite an extensive characterization of the relation of amyloid plaques with microglia, little is known about the initiation of this interaction. In this study, the detailed investigation of very small plaques in brain slices in AD transgenic mice of the line APP-PS1(dE9) revealed different levels of microglia recruitment. Analysing plaques with a diameter of up to 10 μm we find that only the half are associated with clear morphologically activated microglia. Utilizing *in vivo* imaging of new appearing amyloid plaques in double-transgenic APP-PS1(dE9)xCX3CR1^+/-^ mice further characterized the dynamic of morphological microglia activation. We observed no correlation of morphological microglia activation and plaque volume or plaque lifetime. Taken together, our results demonstrate a very prominent variation in size as well as in lifetime of new plaques relative to the state of microglia reaction. These observations might question the existing view that amyloid deposits by themselves are sufficient to attract and activate microglia *in vivo*.

## Introduction

Microglia activation is a characteristic feature of Alzheimer’s disease (AD). Although microglia cells have been found to contact neurofibrillary tangles in the brain of Alzheimer’s patients ([1]), the association of microglia to amyloid plaques is a more frequent and distinguished event ([2]; [3]). Plaques are extracellular proteinaceous aggregates mainly composed of the β-amyloid peptide (Aβ). Proteolytically derived Aβ monomers successively aggregate into oligomeric species and thence amyloid fibrils, ultimately forming the pathognomonic amyloid plaques which gradually grow in size ([4]). In the vicinity of amyloid plaques neurons are damaged, as apparent by dystrophic neurites, the progressive loss of dendritic spines as well as the loss of axons and neurons ([5]; [6]; [7]; [8]).

Microglia are the resident immune cells of the brain actively surveying the brain parenchyma with their fine processes. After brain injury or immunological challenges, microglia are activated leading to the upregulated expression of immune-related molecules, including interleukin-1 (IL-1) and transforming growth factor β ([9]; [10]). Once activated, microglia prominently change their morphology: the ramified processes swell and withdraw, whilst their cell bodies enlarge ([11]). In the AD brain, activation of microglia and clustering around amyloid deposits tend to occur early in the development of plaque pathology. Nevertheless, little is known about the initiating event of this interaction. Here, we specifically focus on very small newly-formed amyloid plaques and attempt to characterize the process by which microglia are attracted to these lesions.

## Material and Methods

### Mice

Mouse lines APP-PS1(dE9) ([12]), CX3CR1-GFP ([13]) and YFP-H ([14]) were purchased from The Jackson Laboratory, Bar Harbor, USA. Briefly, the transgenic APP-PS1 (dE9) mouse line expresses human APP with Swedish mutation and mutant human Presinilin1 (PS1 delta E9) both under the control of the mouse prion protein promoter resulting in abundant amyloid plaques in cortex and hippocampus[12]. The knockin of gfp to the Cx3cr1 locus in CX3CR1-GFP mice results in a GFP-labeling of microglia[13]. In YFP-H mouse line the YFP is expressed under control of Thy1 promoter which leads to a sparse labeling of pyramidal neurons in cortex and hippocampus [14]. Mice were housed in standard cages with food and water ad libitum. The APP-PS1(dE9) line was crossed with CX3CR1-GFP. The resulting offspring [APP-PS1(dE9)xCX3CR1^+/-^] was inbred to generate the [APP-PS1(dE9)xCX3CR1^+/-^], used for in vivo imaging experiments. For control experiments (S1 Fig.) [APP-PS1(dE9)xCX3CR1^+/-^] x [APP-PS1(dE9)xCX3CR1^+/-^] crossings resulted in [APP-PS1(dE9)xCX3CR1^+/+^], [APP-PS1(dE9)xCX3CR1^+/-^] and [APP-PS1(dE9)xCX3CR1^-/-^]. Furthermore APP-PS1(dE9) was crossed to the YFP-H line resulting in [APP-PS1(dE9)xYFP-H] mice, which were inbred. Mice of mixed gender were used for experiments at indicated age.

The studies were carried out in accordance with an animal protocol approved by the Ludwig-Maximilians-University Munich and the government of Upper Bavaria (Az. 55.2–1–54–2531–188–09). The cranial window preparation and *in vivo* imaging were performed under anesthesia, and all efforts were made to minimize suffering of the animals.

### Immunohistochemistry

Briefly, mice anesthetized with an intraperitoneal injection of ketamine/xylazine (0.14 mg ketamine / 0.01 mg xylazine per gram body weight; WDT/Bayer Health Care) were transcardially perfused with PBS and 4% PFA and brains were prepared. 100 μm thick sections were cut from postfixed brains. Free-floating sections were permeabilized with 2% Triton X-100 overnight and blocked with 10% normal goat serum and BSA. Microglia were immunohistochemically labeled using Iba1-antibody (Wako, 1:200; secondary antibody A-21244 by Invitrogen, 1:500). Fibrillar Aβ plaque staining was performed with 145 μM Methoxy-X04 (provided by Prof. Boris Schmidt from TU Darmstadt, Germany) in PBS for 30 min and subsequently washed with PBS. Slices of CX3CR1^-/-^ and CX3CR1^+/-^ mice were bleached before staining for Iba-1 and Methoxy-X04. Fluorescence images were acquired with a confocal laser scanning microscope mounted on an inverted microscope support (LSM 510, Carl Zeiss). Images were acquired 0.22 μm pixel size and frame distance of 2 μm using a 40x/1.3 DIC oil objective. Images were aquired from cortical or hippocampal tissue. Image stacks were acquired spanning a total depth of at least 10 μm above and 10 μm below each selected amyloid plaque.

### Cranial window preparation and in vivo two-photon microscopy

Mice were anesthetized with an intraperitoneal injection of ketamine/xylazine (0.14 mg ketamine / 0.01 mg xylazine per gram body weight; WDT/Bayer Health Care). Additionally, a single dosage of dexamethasone (6 μg/g body weight; Sigma) was intraperitoneally administered immediately before surgery. Utilizing the open skull preparation a cranial window was placed above the somatosensory cortex. For repositioning during repetitive imaging a small metal bar, containing a hole for a screw, was glued next to the window. Directly after surgery mice received subcutaneously a single analgesic treatment with carprophen (7.5 μg/g body weight; Pfizer) and a single antibiotic treatment with cefotaxim (0.25 mg/g body weight; Pharmore).

Two-photon *in vivo* imaging was started after a 3–4 week recovery period post-surgery, utilizing a LSM 5MP setup (Carl Zeiss) equipped with a MaiTai laser (Spectra Physics) and a 20x water-immersion objective (1.0 NA) (Carl Zeiss). For amyloid plaque staining, methoxy-X04 (0.4 to 2.4 mg/kg; provided by Prof. Boris Schmidt from TU Darmstadt, Germany) was intraperitoneally injected 24 hours before imaging ([15]). Methoxy-X04 is a derivate of Congo red, that specifically binds to amyloid deposits in post mortem tissue and *in vivo* and therefore provides a fluorescent label [15]. Mice were anesthetized by Isofluoran. Imaging sessions lasted for no longer than 60 min and laser power was kept below 50 mW to avoid phototoxic effects. We imaged up to three cortical positions with each position spanning 425 μm x 425 μm x 201 μm in x-y-z (pixel distance in x/y: 0.42 μm; pixel distance in z: 3 μm). Care was taken to ensure similar fluorescence levels in space and in time at every imaging session.

### Data analysis

Analysis of confocal and multiphoton images was performed with unprocessed data. From multiphoton imaging data only amyloid plaques were selected for analysis when at all imaging sessions a total image stack with at least 10 μm above and 10 μm below the plaque was recorded. All interaction analysis of microglia with amyloid plaques was performed with 3D data sets. For plaque volume determination, smallest and largest diameter of each plaque was measured from image projections using ZEN software (Zeiss). Assuming the plaque as a spheric element, the volume was calculated using the corresponding mean diameter. For illustration purposes multiphoton images were deconvolved (AutoQuantX2, Media Cybernetics) and filtered with an edge-preserving algorithm, followed by a local contrast change (Imaris 5.0.1, Bitplane). Confocal and multiphoton image stacks were aligned with Imaris 5.0.1, Bitplane.

Statistics: For histological ex vivo analysis a total of 188 small plaques from five animals were identified (54/55/24/23/32). All images were analysed independently and identically. Two photon in vivo imaging revealed a total of 21 new appearing plaques in four animals (2/4/9/6). All time series data points were analysed independently and identically.

## Results

**Fig 1:**
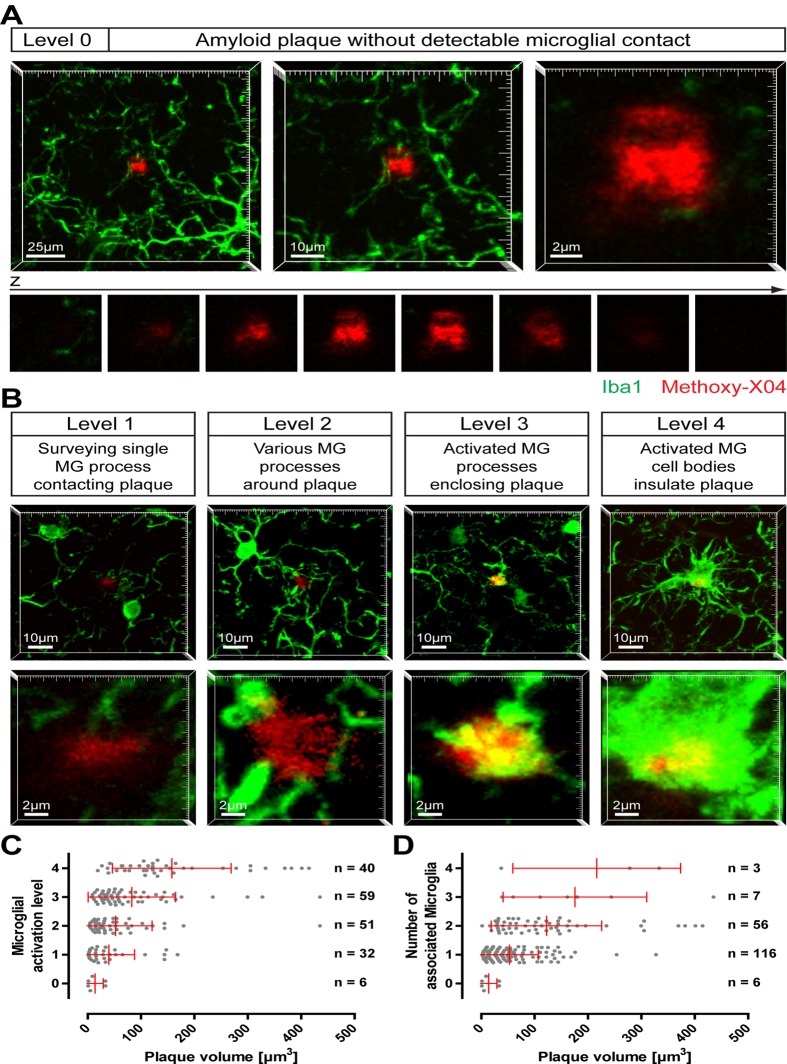
Activation of microglia by small amyloid plaques. On brain slices of 12-month- old APP-PS1(dE9) mice microglial cells were immunohistochemically labelled for Iba1 (green) and amyloid plaques were stained with Methoxy-X04 (red). Small amyloid plaques were imaged and classified according to the microglial status. **A** Image projections of an amyloid plaque lacking any microglial contact (level 0) at three magnifications. Below, the single x-y-planes clarifying that no microglial process touches the plaque. **B** Exemplary images of the stages of plaque-associated microglial (MG) activation (level 1 to level 4). The upper lower magnification illustrates the microglial environment around the plaque, the higher magnification below highlights the individual plaque. Notice that, although each plaque has a similar size, microglial reaction is diverse. **C** Small plaques were classified according to their microglial activation level and plotted against the plaque volume. **D** The number of microglial cells associated to the individual plaques was plotted against the plaque volume. (The brains of five animals were analysed with a total of 188 small plaques. Red bars show mean with standard deviation.)

On brain slices of 12-month-old APP-PS1(dE9) mice, microglia cells were immunohistologically stained and fibrillar amyloid deposits were labelled with Methoxy-XO4. Rarely, we observed small amyloid plaques that were devoid of any detectable microglial contact (Fig. 1A); in most cases, similar sized plaques were clearly contacted by microglia, partly exhibiting an activated appearance (Fig. 1B). To characterize the relationship between plaque size and microglial activation, we specifically imaged small amyloid plaques (which are supposed to correspond to novel plaques) in cortex or hippocampus. Therefore, plaques with a diameter of up to ~10 μm (equates a volume of 524 μm^3^) were chosen randomly, while the microglia channel was not visible to the investigator. Image-stacks were recorded by confocal microscopy covering the total plaque volume with additional 10 μm beneath and above each plaque. Subsequently, the microglial status around the plaque was classified into five levels: (0) Amyloid plaque devoid of any detectable microglial contact; (1) Amyloid plaque contacted by a single surveying microglial process; (2) Amyloid plaque surrounded by various microglial processes; (3) Amyloid plaque enclosed by activated (explicitly swollen) microglial processes; (4) Amyloid plaque insulated from neuropil by activated microglial cell bodies (Fig. 1A-B). Whereas level 3 and level 4 exhibit distinct morphological signs of microglia activation, at level 2 the microglia start to recognize and orientate toward the plaque. At level 0 and level 1, shape and number of microglia processes at and nearby the amyloid plaque are indistinguishable from plaque-free brain areas. It should be noted that all plaques shown in Fig. 1 A-B are of comparable size. Hereunder, we will refer to activated microglia cells based on their morphological appearance (level 3 and level 4).

In total, 188 small amyloid plaques were investigated from the brains of five animals. Six very small plaques (volume between 0.9 and 33.5 μm^3^) were lacking any microglial detectable contact (level 0) (Fig. 1C). An additional 32 plaques were classified as level 1, with volumes reaching up to 168.3 μm^3^ while 40 plaques were classified as level 2 (volume between 3.6 and 143.8 μm^3^). About 50% of small amyloid plaques were associated with clear morphological signs of microglia activation (level 3 and 4; Fig. 1C). The recruitment and activation of microglial cells correlate weakly with plaque size (R^2^ = 0.1040). For example 73% of plaques in level 3 are smaller than 100 μm^3^, 12% are even smaller than 20 μm^3^. Analyzing the number of microglia, we found that most small amyloid plaques were associated with only one microglial cell (Fig. 1D). Only a very tiny quantity of small plaques were surrounded by three or four microglia, with the volume of these plaques ranging from 37.4 μm^3^ to 434.9 μm^3^ (Fig. 1D). These results indicate that small plaques present a variable recruitment and activation of microglial cells around them.

To confirm the *ex vivo* data and explore the dynamic of microglia activation by amyloid plaque formation we utilized *in vivo* two-photon microscopy. Therefore, we crossbred APP-PS1(dE9) mice with CX3CR1 heterozygous knockout mice, which carry the EGFP gene at the CX3CR1 locus providing a specific fluorescent labelling of microglia in the brain. Although previous studies have shown an effect of heterozygous and homozygous knockout of CX3CR1 on amyloid plaque burden in Alzheimer-transgenic mice ([16]; [17]), in our hands plaque density, plaque area, plaque size and microglia area were indistinguishable in cortex and hippocampus of APP-PS1(dE9) and APP-PS1(dE9)xCX3CR1^+/-^ (S1 Fig.).

**Fig 2:**
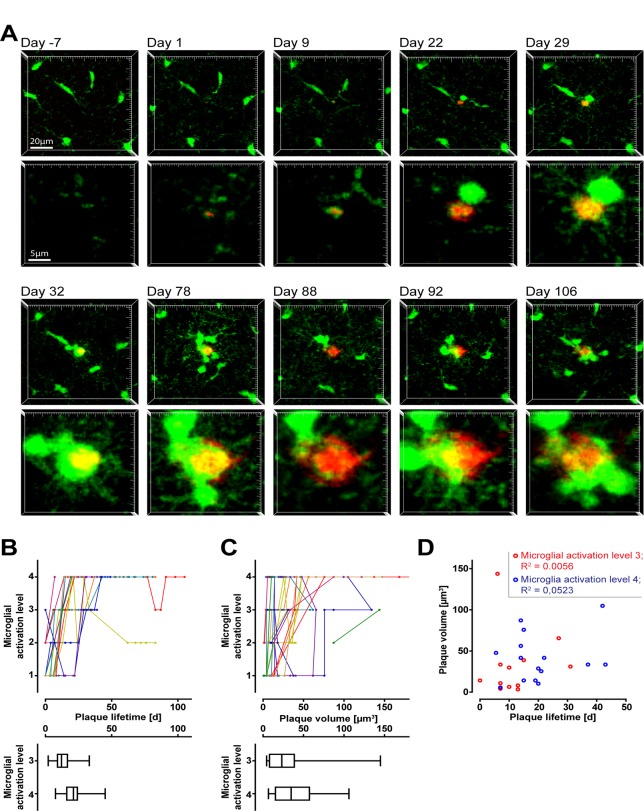
*In vivo* monitoring of amyloid plaque induced microglial activation. In the cortex of PS1(dE9)xCX3CR1^+/-^ mice, individual new appearing plaques (red, Methoxy-X04 stained) and the subsequent microglia activation (green) were followed using two-photon microscopy. **A** Time series of image projections of a newly formed plaque (starting with day 1) and the corresponding microglial reaction. The upper row indicates the microglial environment, the lower row shows the plaque in higher magnification. Notice, at day 1 a microglial process is already contacting the plaque (level 1). At day 9 this plaque was classified as level 3 and level 4 was reached at day 29. Interestingly, at day 88 the plaque is mainly liberated from enclosing microglia, which at day 92 is reverted to level 4 again. **B** Time dependent development of microglial activation level for each plaque (colour coded) beginning with the time point of appearance. The box plots below indicate the distribution of initial time points when microglia activation was reached (level 3 and 4). **C** Size dependent development of microglial activation for individual plaques (colour coded). The box plots below indicate the distribution of initial volume when microglia activation was reached (level 3 and 4). **D** No correlation between plaque lifetime and plaque volume when microglial activation level 3 or 4 are reached.

Starting at an age of six months we repetitively imaged superficial volumes in the somatosensory and the primary motor cortex of APP-PS1(dE9)xCX3CR1^+/-^ mice to scan for newborn, Methoxy-X04 labelled amyloid plaques. To distinguish very small plaques from autofluorescent spots we recorded additionally to the Methoxy-X04 emission (short pass 485 nm) nonspecific emission at 590–650 nm (S2 Fig.). New amyloid plaques were identified as Methoxy-X04 specifically-labelled structures that remained in place and typically grew in size over time (S3 Fig.). In four mice, we were able to detect 21 new appearing plaques in total and followed the microglial reaction on these plaques for up to 106 days (Fig. 2A). Interestingly, at the imaging time-point of first appearance out of the 21 new appearing plaques we could not observe a single plaques without any detectable microglial contact (level 0) (Fig. 2B). Except for one plaque, at the first detection time point all plaques were classified as level 1 or level 2, lacking clear signs of microglial activation. Over time, most plaques induced a more serious morphological activation of microglia, typically terminating at level 4. Nevertheless, the time point when microglial activation was reached differed for individual plaques, ranging for level 4 between 7 and 42 days after first detection (Fig. 2B). Similarly, the plaque volume for microglial activation was different, with the smallest plaque inducing level 4 at a size of 10.3 μm^3^ and the largest at a size of 104.8 μm^3^ (Fig. 2C). There was no correlation between plaque volume and plaque lifetime at the time point, when microglial activation level 3 as well as level 4 was reached (Fig. 2D). Occasionally we also observed a deactivation of microglia from a high level to a lower one (Fig. 2A-C). Together these results suggest that the formation of individual amyloid plaques precedes the activation of microgial cells. Furthermore, the recruitment of microglia does not seem to be directly dependent on the lifetime or the initial size of the corresponding plaque. Although, eventually, all amyloid plaques are surrounded by activated microglial cells (level 3–4), there is substantial variability between individual plaques before reaching this level.

## Discussion

In the brain of AD patients, the amyloid burden is accompanied by clustering of activated microglia around these amyloid plaques. The role of microglia in this pathology is controversial. Hypotheses range from clearance of Aβ-aggregates to potential negative inflammatory effects and neuron elimination mediated by microglia ([18]; [19]; [20]). In this descriptive study, but the first of its kind, we aimed to analyse the initiation of microglial interaction with amyloid plaques in APP-PS1(dE9) transgenic mouse line. For very small plaques we distinguished five levels of increasing interaction in brain slices, four of which could be confirmed by *in vivo* two-photon microscopy. We observed that the degree of microglia reaction showed a great variation with regards to size as well as lifetime of individual small plaques.

In a more detailed description, we therefore herewith confirm the original finding by Meyer-Luehmann and colleagues that the formation of amyloid plaques precedes the recruitment of microglia ([21]). Typically, the plaque is touched by fine microglial processes before swollen processes and finally activated microglial cell bodies envelope the whole plaque. However by the data it cannot be ruled out, that microglia might be involved in the formation of plaques by releasing certain factors into the microenvironment.

An important question is whether the amyloid deposit itself is sensed by the microglia and induces the subsequent microglial reactions. It has been shown that fibrillar Aβ can directly activate microglia IL-1 expression ([22]). Nonetheless, it is not clear why the here observed reaction of microglia to the individual small plaques is so diverse. After emergence of a plaque, we observed that the morphological activation of microglia takes between 7–42 days to start. The variation in initiation sizes and initiation time points after plaque formation of unequivocal microglial activation supports the hypothesis that secondary consequences of amyloid deposition lead to the recruitment of microglia. This view is further supported by the fact that indeed sporadically small plaques without any detectable association to microglia have been found. One possible explanation could be that a developing amyloid plaque directly injures at some time point its surrounding neuropil, and the subsequent release of neuron-derived signalling molecules induces the recruitment of nearby microglial cells. Accordingly, neuritic dystrophies which accompany the development of amyloid plaques might represent the site of acute neuronal tissue damage (S4 Fig.). The different degree of neuropil alteration around a plaque over time could explain the different levels of microglial activation, but at a certain point all plaques are surrounded by activated microglia. However, we cannot rule out that neuronal injury might also be a consequence of microglia activation. Although the microglia in humans might respond differently to fibrillar Aβ, in AD brains microglial activation has been shown to progress with the neuropathological stage of the disease as well as with the progression of individual amyloid plaques ([23], [24]). About one fifth of early, non-fibrillar amyloid plaques in AD brains were found not to be associated with microglia, again questioning whether Aβ aggregates *per se* can recruit these cells ([23]). Interestingly, the late-stage dense core plaques showed less microglial association, paralleled by a reduced number of dystrophic neurites ([23]). Presumably, at this final stage the immediate vicinity of the plaque lacks neuronal tissue that can be damaged.

In summary, we have provided a detailed description of the initial interaction of microglia with newly developing amyloid plaques in an AD mouse model. Our results indicate that the appearance of plaques alone might not be the direct cause of microglia recruitment. Alternatively, the progressive tissue alteration around the plaques might be related to the microglia activation. Clearly, the trigger which actually attracts the microglia to the amyloid lesion remains to be identified.

## Supporting Information

S1 Fig Amyloid plaque deposition is comparable in APP-PS1(dE9)x xCX3CR1^+/+^ and APP-PS1(dE9)xCX3CR1^+/-^. Coronal brain sections of 12 months APP-PS1(dE9)xCX3CR1^+/+^(which are in fact APP-PS1(dE9)), APP-PS1(dE9)xCX3CR1^+/-^ and APP-PS1(dE9)xCX3CR1^-/-^ were bleached and posteriorly immunohistochemically stained for microglia (Iba-1, green), amyloid plaques were labelled with Methoxy-X04 (blue). There was no significant difference in plaque density, plaque area, plaque size distribution or microglia area between CX3CR1^+/+^ and CX3CR1^+/-^ Alzheimer-transgenic animals in cortex and hippocampus. (We analysed 5 CX3CR1^+/+^-mice and 6 CX3CR1^+/—^mice with 5 sections per animal. Error bars indicate SEM.) Because of breading problems we were able to generate only one APP-PS1(dE9)xCX3CR1^-/—^mouse, which we separately included into the graphs with dashed lines. Although statistically not testable, plaque burden in this mouse seems to be highly reduced by the CX3CR1-knockout. Data analysis was performed using Imaris 5.0.1, Bitplane. Surface rendering was performed for the various fluorescent channels resulting in the corresponding plaque or microglia area. Plaque density was determined by fluorescent spot detection.
(TIF)

S2 Fig Detection of auto-fluorescent spots. Two-photon excitation of Methoxy-X04 labelled amyloid plaques was performed at 750 nm and the signal was detected using a short pass (SP) 485 nm filter. To exclude false positive fluorescent spots from analysis, we recorded additionally emission at 590–650 nm. These auto-fluorescent spots were found in the neuropil, but also within microglial cells.
(TIF)

S3 Fig Growth of newborn plaques (**A**) The change in plaque volume was followed over the imaging period for each amyloid plaque analysed for microglia activation (Fig. 2). To compare plaques the volume was normalized to 1 for the time point of first appearance. (**B**) To estimate the growth rate data points of approximately weekly distance (+/- 1 day) were combined and plotted over 5 weeks. The slope of linear regression is 0.575 which results in a weekly growth rate of 4.025. (Error bars show SEM.)
(TIF)

S4 Fig Amyloid plaques cause neuritic damage. *In vivo* microscopic time series of image projections of a new appearing plaque (**A**) and a preexisting plaque (**B**) stained with Methoxy-X04 in a APP-PS1(dE9)xYFP-H mouse. In YFP-H mice the fluorescent reporter YFP is expressed under control of a Thy-1 promotor providing to sparse labelling of neurons. While developing the new (**A**) as well as the preexisting (**B**) plaque cause damage of the neuropil, exemplarily apparent as neuritic swellings around the plaques. Notice that in B the neuritic pathology is moderate at day 1, when the plaque has already a reasonable size, but strongly develops over time.
(TIF)
