# Supplementary figures and images for: Fibrillar Amyloid Plaque Formation Precedes Microglial Activation
Source: PLoS One. 2015 Mar 23;10(3):e0119768. doi: 10.1371/journal.pone.0119768 (PMC4370641; doi:10.1371/journal.pone.0119768)

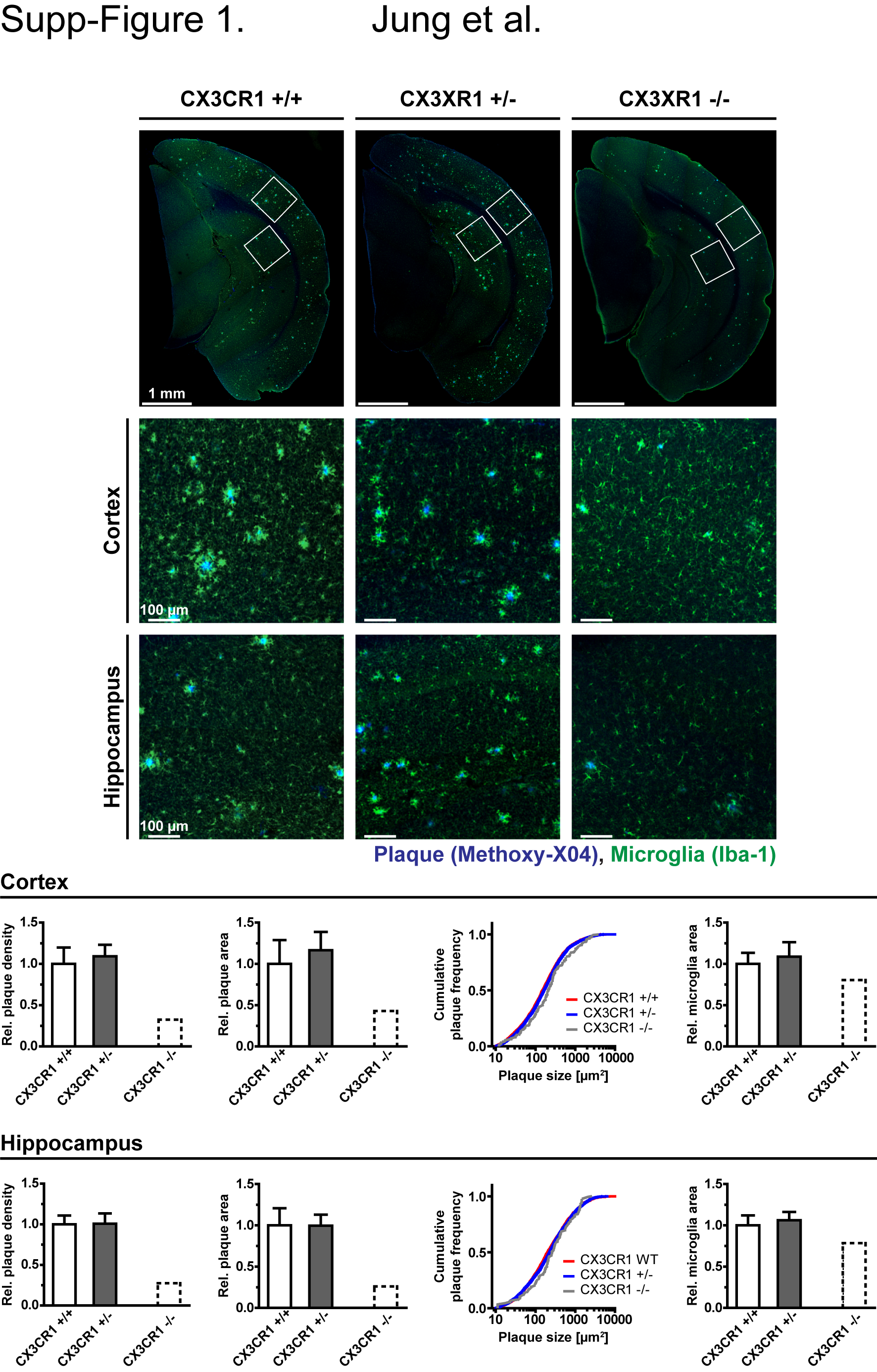

Supplement: S1 Fig — Coronal brain sections of 12 months APP-PS1(dE9)xCX3CR1+/+(which are in fact APP-PS1(dE9)), APP-PS1(dE9)xCX3CR1+/- and APP-PS1(dE9)xCX3CR1-/- were bleached and posteriorly immunohistochemically stained for microglia (Iba-1, green), amyloid plaques were labelled with Methoxy-X04 (blue). There was no significant difference in plaque density, plaque area, plaque size distribution or microglia area between CX3CR1+/+ and CX3CR1+/- Alzheimer-transgenic animals in cortex and hippocampus. (We analysed 5 CX3CR1+/+-mice and 6 CX3CR1+/—mice with 5 sections per animal. Error bars indicate SEM.) Because of breading problems we were able to generate only one APP-PS1(dE9)xCX3CR1-/—mouse, which we separately included into the graphs with dashed lines. Although statistically not testable, plaque burden in this mouse seems to be highly reduced by the CX3CR1-knockout. Data analysis was performed using Imaris 5.0.1, Bitplane. Surface rendering was performed for the various fluorescent channels resulting in the corresponding plaque or microglia area. Plaque density was determined by fluorescent spot detection. (TIF) [file pone.0119768.s001.tif]

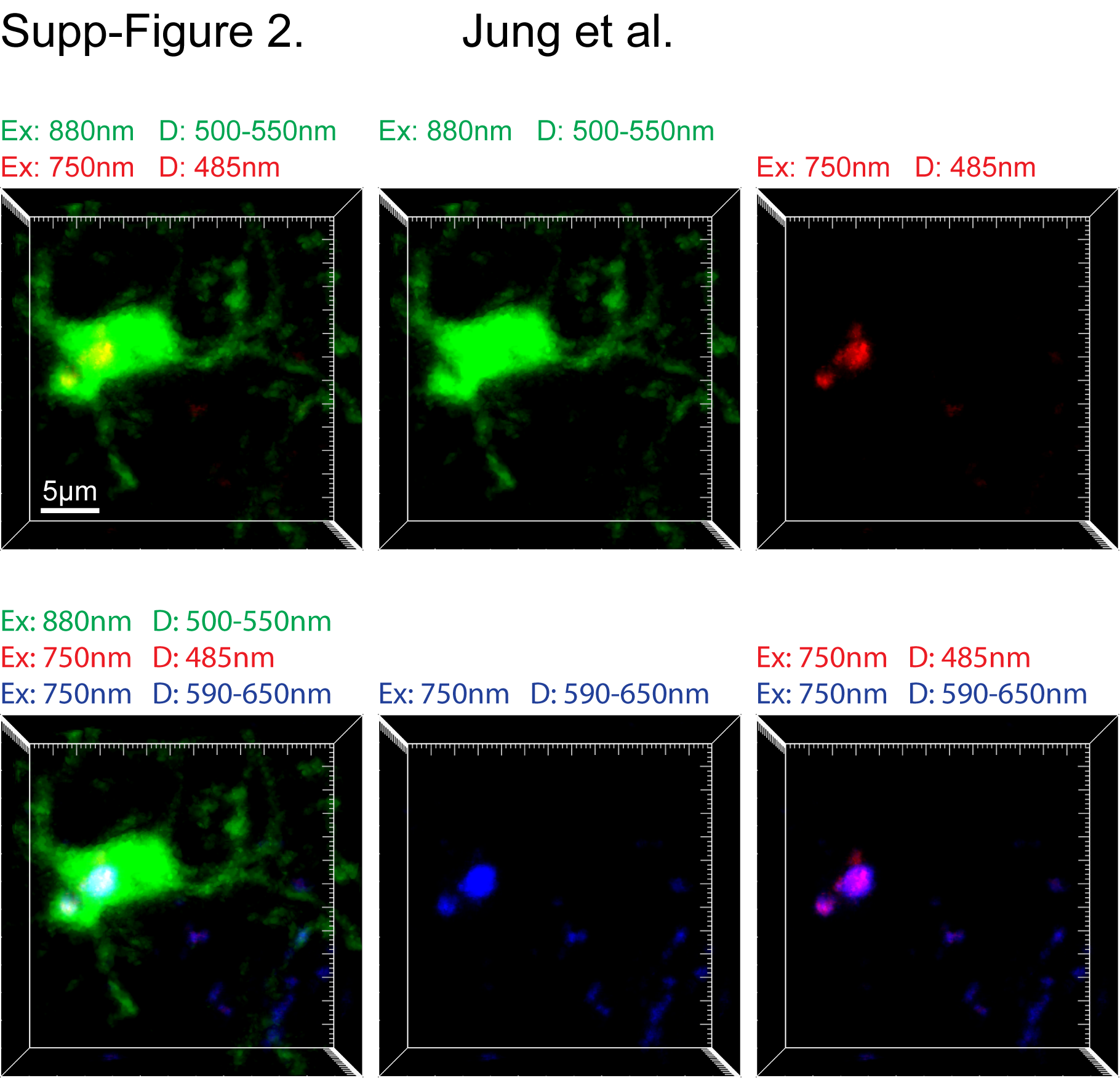

Supplement: S2 Fig — Two-photon excitation of Methoxy-X04 labelled amyloid plaques was performed at 750 nm and the signal was detected using a short pass (SP) 485 nm filter. To exclude false positive fluorescent spots from analysis, we recorded additionally emission at 590–650 nm. These auto-fluorescent spots were found in the neuropil, but also within microglial cells. (TIF) [file pone.0119768.s002.tif]

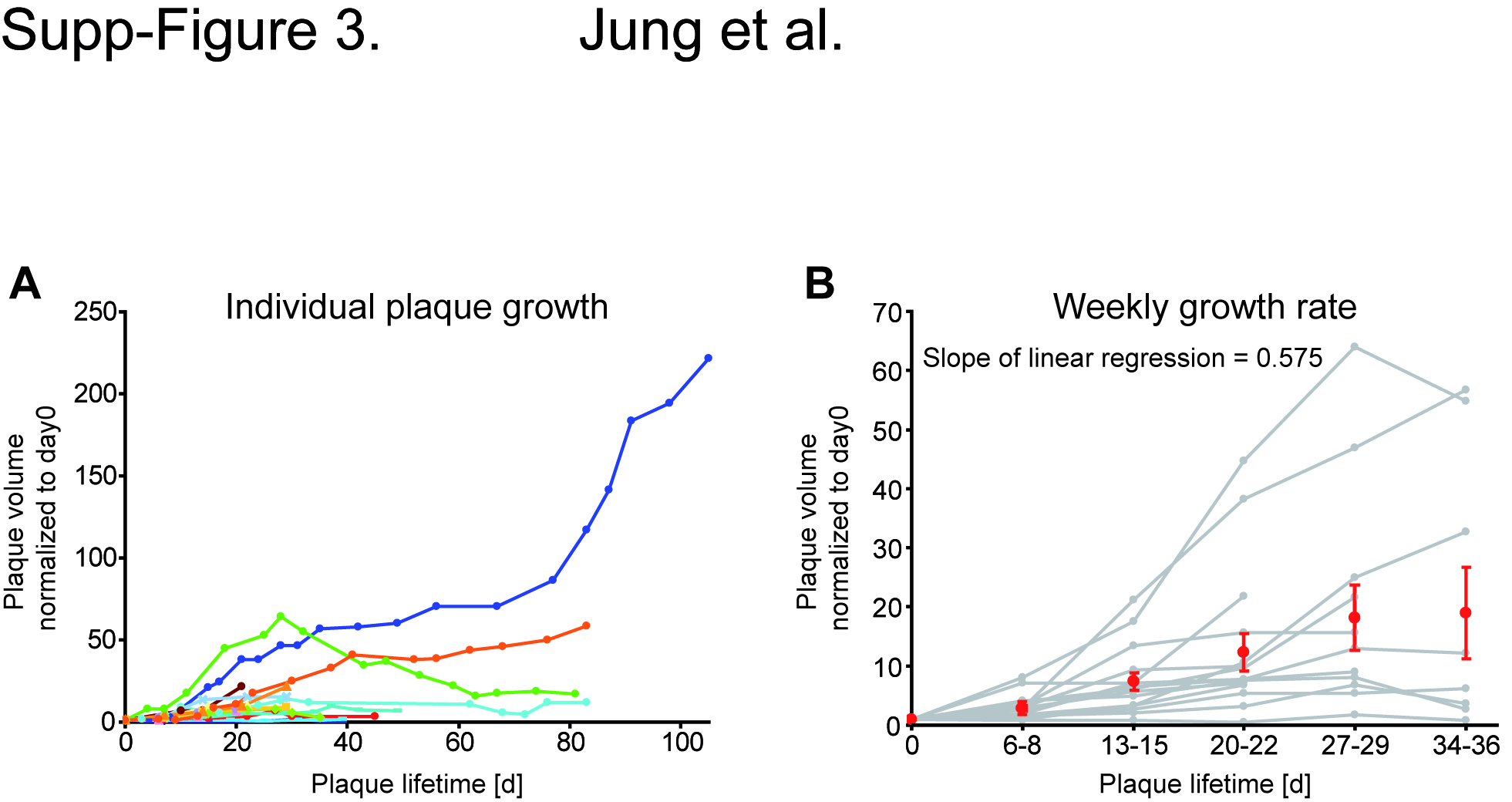

Supplement: S3 Fig — (A) The change in plaque volume was followed over the imaging period for each amyloid plaque analysed for microglia activation (Fig. 2). To compare plaques the volume was normalized to 1 for the time point of first appearance. (B) To estimate the growth rate data points of approximately weekly distance (+/- 1 day) were combined and plotted over 5 weeks. The slope of linear regression is 0.575 which results in a weekly growth rate of 4.025. (Error bars show SEM.) (TIF) [file pone.0119768.s003.tif]

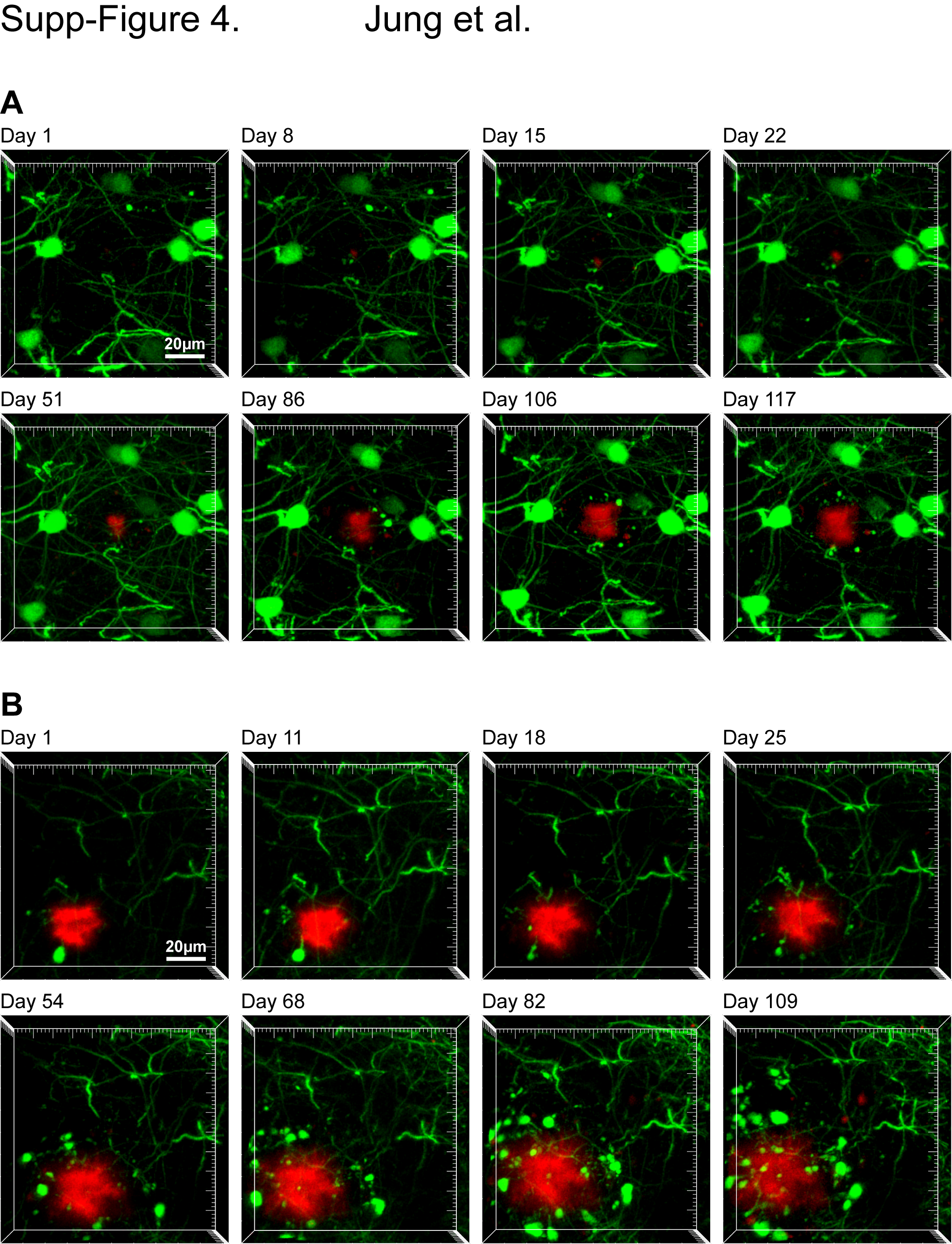

Supplement: S4 Fig — In vivo microscopic time series of image projections of a new appearing plaque (A) and a preexisting plaque (B) stained with Methoxy-X04 in a APP-PS1(dE9)xYFP-H mouse. In YFP-H mice the fluorescent reporter YFP is expressed under control of a Thy-1 promotor providing to sparse labelling of neurons. While developing the new (A) as well as the preexisting (B) plaque cause damage of the neuropil, exemplarily apparent as neuritic swellings around the plaques. Notice that in B the neuritic pathology is moderate at day 1, when the plaque has already a reasonable size, but strongly develops over time. (TIF) [file pone.0119768.s004.tif]
